# Remitting Seronegative Symmetrical Synovitis with Pitting Edema (RS3PE) Syndrome Precedes the Development of Hepatocellular Carcinoma

**DOI:** 10.31662/jmaj.2022-0066

**Published:** 2022-06-17

**Authors:** Sae Ohwada, Noriyuki Akutsu, Yoshiharu Masaki, Shigeru Sasaki, Minoru Nagayama, Yasutoshi Kimura, Ichiro Takemasa, Hiroki Takahashi, Hiroshi Nakase

**Affiliations:** 1 Department of Gastroenterology and Hepatology, Sapporo Medical University School of Medicine, Sapporo, Japan; 2 Department of Surgery, Surgical Oncology and Science, Sapporo Medical University School of Medicine, Sapporo, Japan; 3 Department of Rheumatology, Sapporo Medical University School of Medicine, Sapporo, Japan

**Keywords:** RS3PE, paraneoplastic syndrome, hepatocellular carcinoma

## Abstract

Remitting seronegative symmetrical synovitis with pitting edema (RS3PE) syndrome is characterized by bilateral synovitis and marked pitting edema of the hands and/or feet. Despite the unknown etiology of RS3PE, several reports have described the putative association of this disease with malignant tumors. We herein report the findings of a 76-year-old man with RS3PE syndrome who developed hepatocellular carcinoma 3 years after achieving clinical remission of RS3PE using corticosteroid treatment; high vascular endothelial growth factor and tumor necrosis factor-alpha levels were considered to have contributed to carcinogenesis in this patient. The sequence of clinical events in this case strongly suggests that careful follow-up, even after clinical remission, is necessary for patients with RS3PE syndrome whose malignancy is not confirmed at diagnosis.

## Introduction

Remitting seronegative symmetrical synovitis with pitting edema (RS3PE) syndrome is characterized by rapid-onset symmetrical synovitis; pitting edema over the involved joints, especially the dorsum of the hands; and elevated serum C-reactive protein (CRP) level and erythrocyte sedimentation rate (ESR), but an absence of rheumatoid factor (RF) ^(1)^. It may develop as a paraneoplastic syndrome associated with malignant tumors. Nakashima et al. ^(2)^ reported a case of hepatitis B (HB) virus-related hepatocellular carcinoma (HCC) coexisting at the onset of RS3PE syndrome. However, no previous reports have described HCC occurrence after treatment of RS3PE syndrome. We report the case of an elderly patient diagnosed with HCC three years after the diagnosis of RS3PE syndrome.

## Case Report

**Figure 1.**
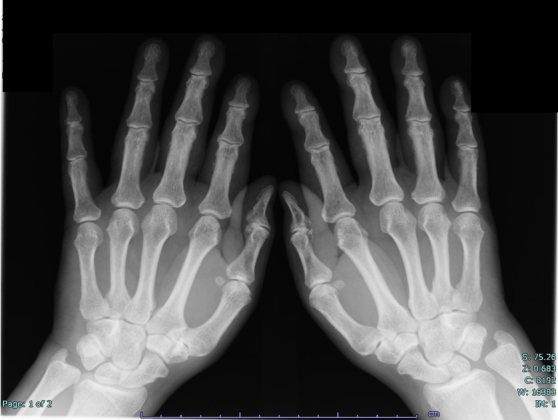
Plain X-ray of both hands shows soft tissue swelling and extensor tenosynovitis without bone erosions.

A 76-year-old man with a month-long history of acute-onset pain in the shoulder, elbow, and back and diffuse edema of the dorsum of the hands was admitted to our institution. He had been receiving diabetes medication for 10 years. Laboratory data indicated elevated CRP levels and ESR, although the RF, antinuclear antibodies, and anti-cyclic citrullinated peptide antibody levels were normal (Table 1). Furthermore, a radiograph of the hand showed synovitis without bone erosion (Figure 1). Upper and lower gastrointestinal endoscopy and contrast-enhanced computed tomography (CECT) showed no abnormalities, including malignancy. He was diagnosed with RS3PE syndrome and received 20 mg/day of prednisolone (PSL), to which he responded well, and the laboratory data subsequently normalized. The dose of PSL was tapered by 2.5-5 mg, and he achieved a steroid-free condition 30 months later.

**Table 1.** Laboratory Data at the Time of Diagnosis.

Laboratory test (Reference value)	Diagnosis of RS3PE syndrome	Diagnosis of HCC
Leukocytes (3900-9800/μL)	3900/μL	5500/μL
Hemoglobin (13.4-17.6 g/dL)	13.6 g/dL	13.8 g/dL
Platelets (15-40 *10^4^/μL)	17.4*10^4^/μL	17.1*10^4^/μL
Albumin (4.1-5.1 g/dL)	2.5 g/dL	3.7 g/dL
Total bilirubin (0.2-1.2 mg/dL)	0.5 mg/dL	0.6 mg/dL
AST (13-30 U/L)	33 U/L	22 U/L
ALT (10-42 U/L)	32 U/L	16 U/L
CRP (0.00-0.30 mg/dL)	9.67 mg/dL	0.24 mg/dL
ESR (0-10 mm/h)	36 mm/h	Not measured
Ferritin (18-250 ng/mL)	503.3 ng/mL	Not measured
PT activation (80%-100%)	85.9%	87.6%
AFP (<10 ng/mL)	Not measured	1.7 ng/mL
PIVKA-II (<40 mAU/mL)	Not measured	23 mAU/mL
IgG (815-1800 mg/dL)	1449 mg/dL	1553 mg/dL
IgM (32-190 mg/dL)	54 mg/dL	88 mg/dL
RF (0-15 IU/mL)	<5 IU/mL	Not measured
ANA (negative)	320	Not measured
Anti-CCP (0.0-4.4 U/mL)	1.0 U/mL	Not measured
PR3-ANCA (0.0-3.4 EU)	<1.0 EU	Not measured
MPO-ANCA (0.0-3.4 EU)	<1.0 EU	Not measured
Hyaluronic acid (≤50.0 ng/mL)	Not measured	56.0 ng/mL
M2BPGi (<1.00)	Not measured	1.27
7S domain of type IV collagen (≤4.4 ng/mL)	Not measured	5.4 ng/mL
Fib-4 index (<1.3 points)	2.41 points	2.48 points

AST, aspartate transaminase; ALT, alanine aminotransferase; CRP, C-reactive protein; ESR, erythrocyte sedimentation rate; PT, prothrombin; AFP, alpha-fetoprotein; PIVKA-II, protein induced by vitamin K absence or antagonist-II; RF, rheumatoid factor; IgG, immunoglobulin G; IgM, immunoglobulin M; ANA, antinuclear antibody; Anti-CCP, anti-cyclic citrullinated peptide; PR3-ANCA, proteinase-3 antineutrophil cytoplasmic antibody; MPO-ANCA, myeloperoxidase antineutrophil cytoplasmic antibody; M2BPGi, Mac-2 binding protein glycan isomer; Fib-4 index, Fibrosis-4 index

**Figure 2.**
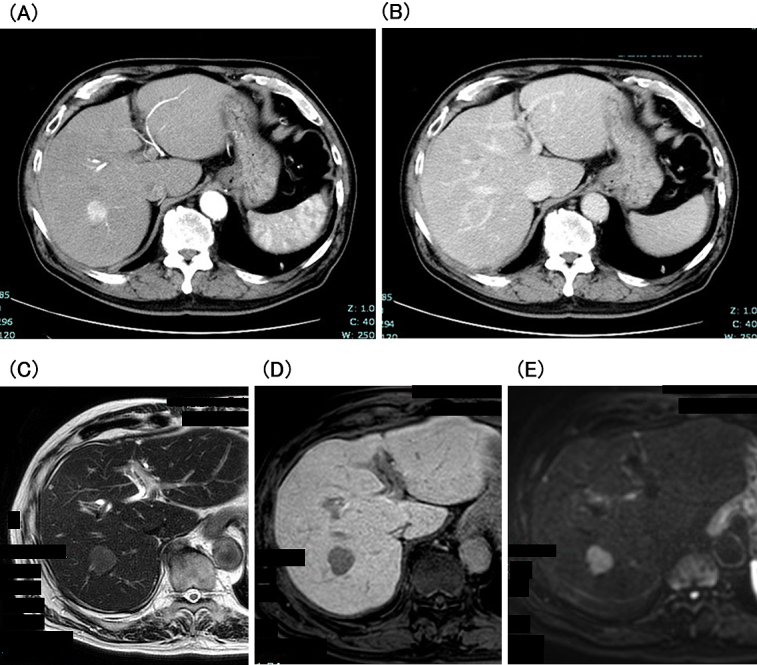
Contrast-enhanced CT shows that the tumor was hyperdense in the arterial phase (A) and hypodense in the delayed phase (B) images. EOB-MRI shows that the tumor was hyperintense on T2WI (C) and DWI (D) and clearly hypointense in the hepatocyte phase (E). EOB-MRI, gadoxetic acid-enhanced magnetic resonance imaging; T2WI, T2-weighted image; DWI, diffusion-weighted imaging.

**Figure 3.**
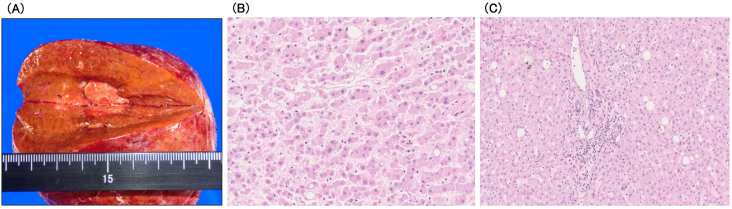
The pathological findings of liver-excised tissues. (A) The resected specimen. No cirrhosis is observed. (B) Moderately differentiated hepatocellular carcinoma (HE ×20). (C) The background liver tissue contained approximately 10% fat and enlargement of the portal tract with mild-to-moderate inflammatory cell infiltration. Bridging fibrosis was not observed (HE ×10).

Three years after that, abdominal ultrasonography (AUS) revealed a 20-mm-diameter hypoechoic mass in segment 7 of the liver. CECT showed that the tumor was hyperdense in arterial phase images and hypodense in delayed phase images. In addition, the tumor showed hyperintensity on diffusion-weighted imaging and was clearly hypointense to the surrounding liver parenchyma in the hepatocyte phase (Figure 2). Based on these findings, the patient was diagnosed with HCC. Laboratory data showed negative results for hepatitis B virus surface antigen and antibody, hepatitis B virus core antibody, and hepatitis C virus antibody. The patient also had no history of alcohol-induced hepatitis. The patient’s body mass index was 26.7. The Child-Pugh status was class A, and the Fib-4 index was 2.48. Vascular endothelial growth factor (VEGF), matrix metalloproteinase-3 (MMP-3), and tumor necrosis factor-α (TNF-α) levels were elevated (Table 2). AUS showed a mildly fatty liver surrounding the tumor. Subsequently, laparoscopic retrohepatic segmentectomy was performed, and the histological diagnosis was moderately differentiated HCC (pT2N0M0, pStage II). Pathologically, the background liver tissue contained approximately 10% fat, and the portal tract showed mild inflammatory cell infiltration. Bridging fibrosis was not observed (Figure 3). The patient is alive without cancer recurrence or RS3PE symptoms three years after the surgery. The MMP-3 level normalized (51.5 ng/mL), whereas serum levels of VEGF (158 pg/mL) and TNF-α (11.7 pg/mL) remained high (Table 2).

**Table 2.** Changes in Serum Levels of VEGF, TNF-α, and MMP-3.

Laboratory test (Reference value)	At the time of diagnosis of RS3PE	At the time of diagnosis of HCC	Post-operation of HCC
VEGF (≤38.3 pg/mL)	Not measured	141 pg/mL	158 pg/mL
MMP (36.9-121 ng/mL)	Not measured	380 ng/mL	51.5 ng/mL
TNF-α (0.75-1.66 pg/mL)	Not measured	10.4 pg/mL	11.7 pg/mL

VEGF, vascular endothelial growth factor; MMP3, matrix metalloproteinase-3; TNF-α, tumor necrosis factor-α

## Discussion

RS3PE syndrome and malignant tumors often coexist simultaneously, but cancer is occasionally diagnosed after the onset of RS3PE syndrome. An interesting point in this case was that HCC was discovered three years after the diagnosis of RS3PE syndrome, even though no apparent malignant tumor was found at that time. Therefore, patients with RS3PE syndrome should be monitored for malignancy for an extended period, even after RS3PE symptoms disappear.

Underlying malignancies may cause RS3PE syndrome through an inflammatory process involving VEGF and TNF-α, especially in paraneoplastic RS3PE syndrome ^(3), (4)^. No reports directly compare VEGF or TNF-α levels between paraneoplastic and non-paraneoplastic RS3PE syndrome. Generally, VEGF levels decrease after treatment in paraneoplastic RS3PE syndrome ^(5)^. Tabeya et al. ^(6)^ reported that the serum levels of VEGF peaked at 572 pg/mL and decreased after glucocorticoid pulse therapy. However, the serum VEGF level did not decrease even after surgery in our patient. Our patient had a fatty liver and diabetes, and the Fib-4 index indicated moderate fibrosis of the liver; it cannot be denied that chronic liver damage may have caused the HCC or chronic liver damage contributed to the elevation of the serum VEGF level. However, HCC rarely developed in patients with a Fib-4 index of <2.67 ^(7)^. Therefore, the inflammation caused by RS3PE syndrome may have a synergistic effect on carcinogenesis. We also observed elevated serum MMP-3 levels at the time of HCC diagnosis. High serum levels of MMP-3 have been reported in patients with RS3PE syndrome related to malignant tumors ^(8)^. Although the immunohistochemical analysis of MMP-3 in the surgical specimen was not performed, the reduction in MMP-3 levels after radical resection indicates that it may be a tumor marker. Further reporting of similar cases is required in the future.

## Article Information

### Conflicts of Interest

None

### Acknowledgement

We would like to thank Editage (www.editage.com) for English language editing.

### Author Contributions

The first draft of the manuscript was written by Sae Ohwada. All authors commented on previous versions of the manuscript. All authors read and approved the final version of the manuscript.

### Informed Consent

Informed consent for the publication of this case report was obtained from the patient
